# Tomographic Absorption Spectroscopy for H_2_O Transport in a Laminar Jet with Inverse Concentration Gradient

**DOI:** 10.3390/s22165939

**Published:** 2022-08-09

**Authors:** Kin-Pang Cheong, Dingfeng Shi, Shaotong Liu, Junjun Wu, Kun Duan, Yong Song, Wei Ren

**Affiliations:** 1 School of Aeronautics and Astronautics, Sichuan University, Chengdu 610065, China; 2 Key Laboratory of Low-Grade Energy Utilization Technologies and Systems and School of Energy and Power Engineering, Chongqing University, Chongqing 400044, China; 3 Department of Mechanical and Automation Engineering, The Chinese University of Hong Kong, Shatin, N.T., Hong Kong SAR, China; 4 Sichuan Aerospace Zhongtian Power Equipment Co., Ltd., Chengdu 610199, China

**Keywords:** tomographic absorption spectroscopy (TAS), tunable diode laser absorption spectroscopy (TDLAS), species transport, diffusion, laminar jet flow

## Abstract

We report a tomographic absorption spectroscopy (TAS) study of water vapor transport in a laminar jet issuing into the ambient. The jet was generated using compressed dry air that was straightened by a honeycomb and a smooth contraction nozzle. A TAS scheme using the water vapor in the ambient as absorbing species and the absorption line near 1368.598 nm was proposed to study the H_2_O transport in the laminar jet with an inverse concentration gradient. One-dimensional tomography was conducted at various heights above the nozzle, and the results were validated by the predictions from computational fluid dynamics (CFD) simulations. Particularly, the variations in the concentration gradient in the shear layer at different heights were captured. The 2D distribution of water concentration in the dry laminar jet was obtained experimentally. The present study shows that TAS has great potential in the research of mass transfer and scalar field of gaseous flows.

## 1. Introduction

The precise sensing of gaseous processes that involve diffusion and transport, in either reactive or non-reactive flows, is important for both scientific research and engineering applications. Particularly, in the context of global warming and atmospheric pollution, the emissions of greenhouse and hazardous gases in industrial production, transportation and propulsion require accurate and long-term monitoring and effective reduction. The emission monitoring can be accomplished by the novel sensing technologies that have emerged from the fast development of laser sensors and detection principles in the past decades [1,2], which obtain the gas states (temperature, concentration, pressure, etc.) by interpreting the spectrum induced by the interaction of gaseous molecules and laser photons. Among them, tunable diode laser absorption spectroscopy (TDLAS) attracts extensive attention in environmental sensing and combustion diagnostics due to its *in situ*, fast and highly quantitative measurement, yet compact and simple optical setups [3,4,5].

TDLAS has promoted combustion research in many ways, including combustion chemistry (uniform condition) [6] and reaction field (non-uniform condition) [7] measurements. The line-of-sight (LOS) nature and fast temporal response of TDLAS make it extremely suitable for extracting the reaction rate parameters from uniform combustion within the shock tube [6]. Unfortunately, this LOS nature also hinders its application in non-uniform flames until it is combined with computed tomography (CT), which resulted in tomographic absorption spectroscopy (TAS). To date, detailed measurements of various flames, from axisymmetric laminar flames [8,9] to turbulent jet engine tail flames [10], from one-dimensional (1D) to three-dimensional (3D) [11,12,13,14], from single parameter of temperature to multi-physicochemical parameters [15,16,17,18], have been demonstrated by TAS. By using hyperspectral absorption tomography, Ma et al. [10] reported the first 2D imaging of temperature and H_2_O concentration fields of the J58 aero-engine with a spatial resolution of 36.3 mm and a temporal resolution up to 50 kHz. Senders et al. [17] investigated the combustion fields of two hybrid rocket injectors by simultaneously measuring the temperature, CO, CO_2_ and H_2_O distributions using TAS, which showed the capability of TAS in combustion performance evaluation and optimization. Wei et al. [13] extended TAS to 3D imaging with the assistance of a high-speed infrared camera, revealing the volumetric distributions of temperature, CO and CO_2_ of two laminar flames by collecting absorption information from limited angles.

Despite its capability in multi-dimensional and multi-parameter measurements, TAS still faces various challenges that have been pointed out by previous studies [19,20,21,22,23,24]. The ill-posed nature of tomography requires regularized spectral reconstruction [19] and a well-arranged setup of beam grid [20]. With the help of machine learning, the reconstructed error during reconstruction can be mitigated [25,26]. It is also found that the reliability and accuracy of TAS depend on the selection of absorption transitions when the target field involves a wide range of temperatures [21,22]. Moreover, although it still needs further investigation, the flow state of the target field is found to have an additional influence on LAS measurement [23,24], which may lead to unexpected errors in TAS. It should be pointed out that the accuracy of spectral parameters in a database such as HITRAN [27] or HITEMP [28] has essential importance to the final results of TAS. In practice, most of these parameters need to be calibrated [29] to ensure a reasonable reconstruction of the unknown distributions [9]. This may be because the complicated interactions between the molecules and line mixing phenomenon at high temperature/high pressure under combustion environment lead to deviations from the standard model that is applied to obtain the spectroscopic parameters. Therefore, it is necessary to decouple the above-mentioned influences on TAS, especially the influence of fluid flow.

To address the above issues, different from previous TAS studies in complex combustion, we built a jet flow TAS platform, which is designed to conduct TAS in a non-reactive jet flow with various conditions in flow state (laminar or turbulent), species composition (single or multiple absorption species), and temperature (room or high temperature). In this way, the influences of these complex conditions under combustion environment can be decoupled and clarified, which might result in a new theory or method of absorption spectroscopy for combustion diagnostics. The present study is one of the serial investigations into advanced laser absorption spectroscopy for flow systems. On the other hand, by applying TAS to non-reactive fluid flow at room temperature, new technologies for fluid research may be developed. To the best of our knowledge, this is the first TAS study on a non-reactive, dry (comparing to the ambient), laminar jet flow at room temperature to explore the applications of TAS in the research of mass transfer and scalar field of gaseous flow. Particularly, we use the environmental H_2_O as the target absorbing species, which is detected by the transition at 1368.598 nm. In this study, a computational fluid dynamics (CFD) simulation is also performed for cross-validation. Based on the near-infrared (NIR) TAS results of the H_2_O concentration distributions at different heights above a laminar jet flow, the 2D contour of H_2_O diffusion with inverse concentration gradient is obtained.

## 2. Methods

### 2.1. Theory

**Figure 1:**
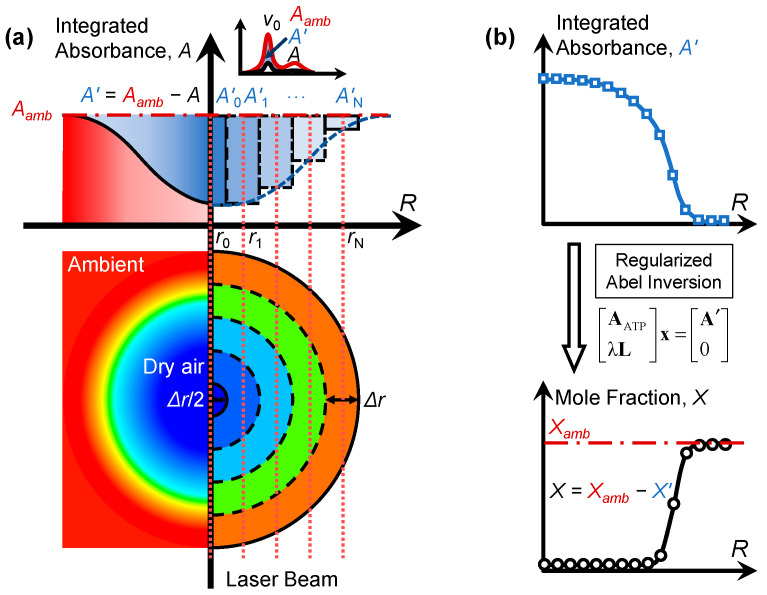
Illustration of the present 1D tomography for a dry, axisymmetric, laminar jet with the inverse H_2_O concentration gradient: (**a**) acquisition of LOS integrated absorbance; (**b**) reconstruction of radial species concentration using Tikhonov-regularized Abel inversion.

Figure 1 illustrates the present 1D tomography for a dry, axisymmetric, laminar jet with the inverse H_2_O concentration gradient, which differs from the TAS of an axisymmetric flame or humid laminar jet with abundant H_2_O molecules. The main difference is the way of extracting integrated absorbance for tomographic reconstruction and the final species concentration. Usually, for axisymmetric flows, the absorption information across the target flow is reconstructed from the measured LOS absorbance along various radial positions. Then, the local temperature or species concentration can be obtained from the reconstructed absorption coefficients using beer’s law. However, the dry laminar jet has little contribution to the LOS integrated absorbance *A* compared to the ambient, as shown in Figure 1. The impact of dry air can be expressed mathematically as:

(1) $${A^{\prime }}_{v0}\left(x\right)=A_{v0,amb}\left(x\right)-A$$

where the subscript *amb* denotes ambient and *v*_0_ denotes the line-center. The integrated absorbance can be calculated by the incident (*I*_0_) to transmitted (*I*_t_) laser intensities using Beer’s law as:

(2) $$A_{v0}\left(x\right)=-\int_{-\infty }^{\infty }\ln{\left(I_{t}(x)/I_{0}(x)\right)}_{\nu }d\nu$$

The impact of dry air can be written by substituting Equation (2) into (1):

(3) $$\begin{array}{ll} {A^{\prime }}_{v0}(x) & =-\left(\int_{-\infty }^{\infty }\ln{\left(I_{t,amb}^{}(x)/I_{0}(x)\right)}_{\nu }d\nu -\int_{-\infty }^{\infty }\ln{\left(I_{t}^{}(x)/I_{0}(x)\right)}_{\nu }d\nu \right)\\ & =-\int_{-\infty }^{\infty }\ln{\left(I_{t,amb}^{}(x)/I_{t}^{}(x)\right)}_{\nu }d\nu \end{array}$$

For *A_v_*_0_ measured with equal spacing as shown in Figure 1, the local absorption coefficient *f*(*r*) can be reconstructed with the Tikhonov-regularized three-point Abel inversion (ATP):

(4) $$\left[\begin{array}{l} A_{\mathrm{ATP}}\\ \lambda L \end{array}\right]x=\left[\begin{array}{l} A^{\prime }\\ 0 \end{array}\right]$$

where **A**_ATP_ is the axisymmetric 1D projection matrix, **A′** = {*A*_0_, *A*_1_, …, *A*_N_}^T^ and **x** = {*f*(*r*_1_), *f*(*r*_2_), …, *f*(*r*_N_)}^T^ are the vectors of the measured integrated absorbance and reconstructed spectrally integrated absorption coefficient at different locations, respectively, λ is the regularization parameter that can be determined by the L-curve method [30], and **L** is a *N* × (*N* + 1) smoothing matrix:

(5) $$L_{ij}=\left\{\begin{array}{ll} 1, & \;\mathrm{if}\;i=j\\ -1, & \;\mathrm{if}\;i=j+1\\ 0, & \;\mathrm{otherwise} \end{array}\right.$$

After reconstructing the local integrated absorption coefficient *f*(*r*), the mole fraction of the absorbing species originally in the ambient air but removed by the dry flow can be derived:

(6) $$X^{\prime }(r)=\frac{f(r)}{PS(T(r))}$$

where *P* is the pressure and *S* is the temperature-dependent linestrength [1]. Finally, the actual mole fraction of the absorbing species in the flow field is:

(7) $$X(r)=X_{amb}-X^{\prime }(r)$$

### 2.2. Experimental Setup

**Figure 2:**
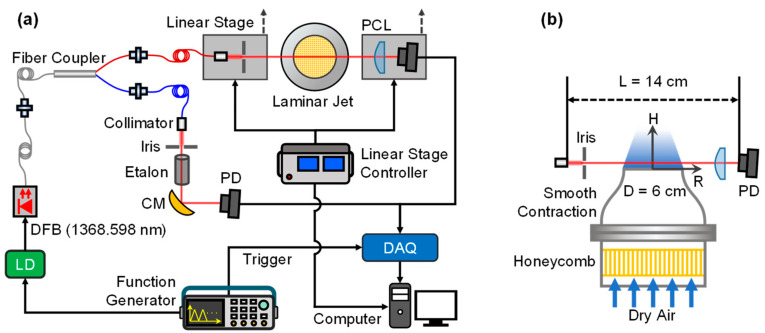
Schematic of the experimental setup: (**a**) optical system for the present study; (**b**) schematic of the nozzle and optical setup. CM, concave mirror; DFB, distributed-feedback laser; LD, laser driver; PCL, plano-convex lens; PD, photodetector; DAQ, data acquisition.

Figure 2 presents the schematic of the present experimental setup for investigating the H_2_O transport in a dry laminar jet with TAS. As shown in Figure 2a, a near-infrared distributed-feedback (DFB) laser with the center wavelength at 1.3686 μm (69sensor, Wuhan) was utilized as the laser source, whose temperature and current were driven by a laser driver (Wavelength Electronics) with the triangle current scanning signal (1 kHz) from a function generator (Tektronix, Beaverton, OR, USA). The laser was then 50:50 split into two beams by a single mode 1 × 2 fused fiber coupler, one for LOS absorption measurement and the other for relative wavenumber determination with the help of a single crystal silicon etalon (FSR = 0.5 GHz). After collimation, the power of the laser beam was reduced by an iris to avoid saturation of the NIR photodetector (Thorlabs, Newton, NJ, USA). A dual-channel DAQ card (NI, PCI-6115) with a sampling rate of 10 MHz per channel was utilized for data acquisition. To reduce the disturbance of the flow while maintaining the significance of the absorption signal, the laser collimator and the photodetector were placed at a distance *L* = 14 cm, and they were installed, respectively, on two motorized linear stages (uncertainty ± 0.005 mm) and controlled simultaneously by identical preset routine. The radial stepping resolution for the present TAS measurement was Δ*r* = 1 mm to capture the H_2_O gradient within the shear layer effectively.

Figure 2b provides the details of the laminar jet nozzle. Compressed air from gas cylinders (purity of 99.8%) was controlled by mass flow controllers (Sevenstar Co., Beijing, uncertainty ± 0.5%) with a flow rate of 60 L/min. Before injecting vertically into the air, the dry air flow went through a honeycomb straightener and a smooth contraction nozzle to mitigate the effects of non-uniform flow and boundary layer on the laminar jet. The smooth contracting part was 3D printed by stereo lithography appearance (SLA) with a diameter of 60 mm and a 5 mm-thick wall. The curve of the nozzle was similar to that by Bouvet et al. [31]. Under these flow conditions, the Reynolds number of the laminar flow *Re* was 1317, which was selected after trials. It was found that the laminar jet was prone to be disturbed by the ambient convection when *Re* was less than 1000, while the flow state would become turbulent when *Re* was too high. In addition, an exhaust hood was installed above the laminar jet to provide coflow for stabilization. The velocity of the ambient flow induced by the exhaust hood was estimated to be around 0.1 m/s.

During the absorption measurement, the room temperature and relative humidity were monitored by an electronic thermohydrometer (±2.5%) and the readings remained 30.8 °C and 65%, respectively. Each TAS measurement at a specific height above the nozzle *H* took about 4 min and the measurements were conducted from *H* = 1 mm to 10 mm with a resolution Δ*H* = 1 mm for the present laminar jet; thus, a complete measurement took about 40 min in total. Temperature *T* and pressure *P* were considered constant. Small variations in these two parameters had little impact on the measurement. Uncertainty analysis is provided in the next section.

**Figure 3:**
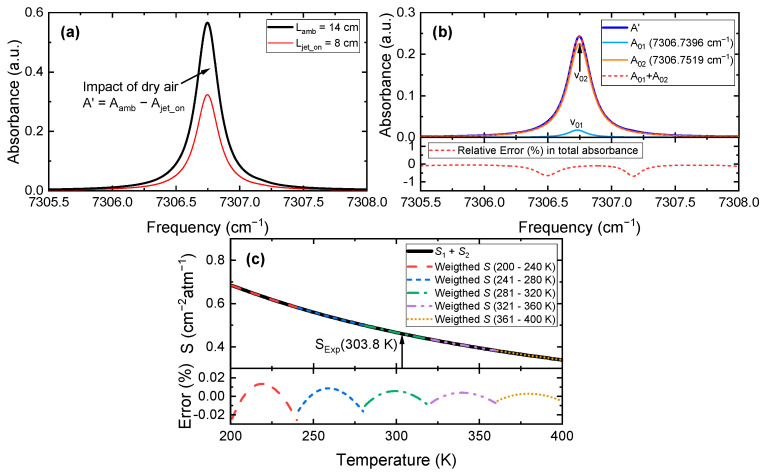
Simulated absorbance and linestrength of the selected transitions at typical measurement conditions: (**a**) LOS absorbances of the ambient and central position of the laminar jet; (**b**) absorbances of selected main transitions and their summation compared to the total absorbance; (**c**) merged linestrength by segmental fitting.

The selection of absorption transition is important for TAS measurements [22]. In the present study, two NIR absorption transitions near 1368.598 nm were selected, whose spectroscopic parameters (frequency, wavelength, linestrength *S* and lower state energy *E*′′) are listed in Table 1. Figure 3a plots the simulated LOS absorbance of the selected transitions at typical measurement conditions *P* = 1 atm, *T* = 303.3 K, *X*_H2O_ = 0.03, *L*_amb_ = 14 cm (ambient), *L_jet_on_* = 8 cm (laminar jet on) based on the HITRAN2020 database [27]. By subtracting the LOS absorbance when the laminar jet is on, the impact of dry air can be obtained, as shown in Figure 3b. It is demonstrated that the absorption lines at 7306.7396 cm^−1^ and 7306.7519 cm^−1^ predominated the contribution to the total absorbance; thus, they are selected for the present study. The linestrengths of these two main absorption lines were merged into a single line by segmental fitting following Wang et al. [32], as displayed in Figure 3c.

It is worth noting that the above optical setup allows the measurement of the species transport in a laminar jet flow by using the ambient H_2_O molecules as the absorption indicator at room temperature, which may be extended to asymmetric or turbulent flow research as a new technique for the study of scalar transport and mixing of flow fields.

### 2.3. CFD Simulation

**Figure 4:**
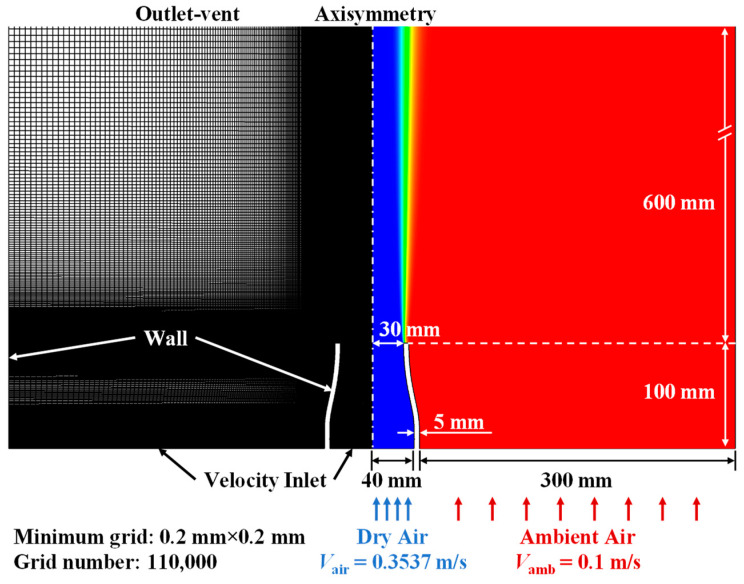
Numerical setup for the CFD simulation of the present laminar jet.

CFD simulation was performed for cross-validation, with the computational domain and boundary condition setups illustrated in Figure 4. The computational domain was extended to the size of 345 mm × 700 mm for simulating the laminar jet injected into the open air. By considering the axisymmetric geometry of the present flow problem, 2D axisymmetric domain and solvers were applied. The minimum grid size was set as 0.2 mm × 0.2 mm in jet core and shear layer regions with the non-uniformly growing grid in radial and downstream directions, resulting in about 110,000 cells in total. The finer grid was also tested but the variations in the result were less than 1%. Uniform velocity inlets were set for dry air and ambient air boundaries with a velocity of *V*_air_ = 0.3537 m/s and *V*_air_ = 0.1 m/s, respectively, and the corresponding H_2_O mole fractions were set as 0.002 and 0.02889 according to the composition of compressed dry air and the reading of thermohydrometer. The radial boundary was set as the wall for better convergence. The kinetic-theory model was used for mass diffusivity in the species transport model. The thermodynamic and transport data for all the species were imported from the NIST database [33]. All the terms in the governing equations were second-order-discretized and the convergence criteria were 10^−6^ for all equations. The present numerical simulation was conducted using ANSYS Fluent in a 16-core workstation.

## 3. Results and Discussion

**Figure 5:**
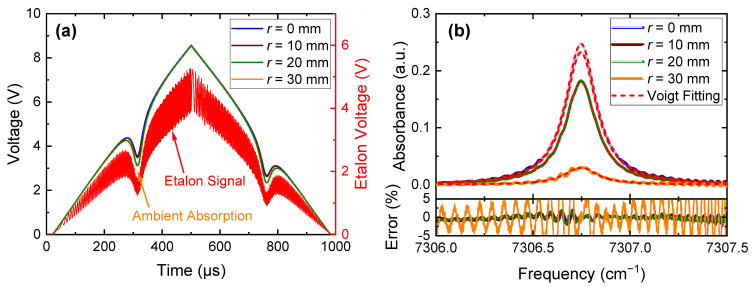
Typical signals and data processing for the present study: (**a**) typical raw signals and etalon signal; (**b**) absorbance and Voigt fitting at different locations, also shown are the relative errors of the fitting. These typical data are collected at *H* = 1 mm.

The typical raw data and etalon signal for temporal to frequency transformation are shown in Figure 5a, which were collected at *H* = 1 mm. To eliminate the white noise from various devices, we obtained the mean signals by averaging 100 scans, resulting in an effective temporal response of 0.1 s. By using the ambient absorption signal at *r* = 45 mm as the baseline, the spectral absorbances at different locations were calculated. Some representative results together with their Voigt fittings are provided in Figure 5b. It can be seen from Figure 5b that the fitting errors are generally less than 2.5%, while some wave-like signals are observed in the absorbance signals, although the raw signals are quite smooth after averaging in Figure 5a. These wave-like signals may result from the intrinsic characteristics of the NIR DFB laser or the photodetector, or the noise from other heavy-duty devices in the laboratory. A low-pass filter with cutting frequency of 8000 Hz or averaging over more scans will mitigate these wave-like signals. However, it might result in either a change in the line shape or an increase in the experiment time. Further investigation and additional actions are needed to better mitigate this problem.

**Figure 6:**
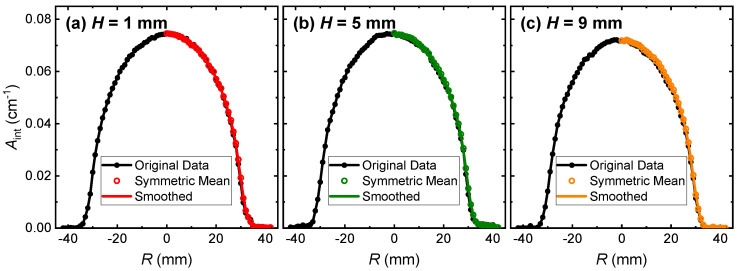
LOS integrated absorbance *A*_int_ at different heights above the nozzle.

The LOS integrated absorbance *A*_int_ can be derived from the non-linear Voigt fitting process. To ensure the axisymmetry of the laminar jet, i.e., that the laminar jet was not disturbed during the measurement, the laser beam was moved from *R* = −42 mm to 42 mm to cover the nozzle and the ambient. Note that if the laminar jet is significantly disturbed by the ambient, then the distribution of *A*_int_ along the radial direction would be distorted away from symmetry. Typical distributions of *A*_int_ at different *H* above the nozzle are provided in Figure 6. It is shown that the original data of the measured *A*_int_ follow the symmetric distribution quite well, demonstrating the laminar jet was steady during the experiment, although there was a slight deviation from symmetry. Therefore, we took two additional steps for *A*_int_ before the tomographic reconstruction to reduce the uncertainty during the Abel inversion: (1) the symmetric mean of *A*_int_ was evaluated by averaging *A*_int_ at the same distance away from the central axis; (2) the symmetric mean of *A*_int_ was then smoothed using the ‘sgolay’ function (the Savitzky–Golay filter) with a window size of eight data points. Together with the Tikhonov regularization, the reconstruction uncertainty can be effectively suppressed.

**Figure 7:**
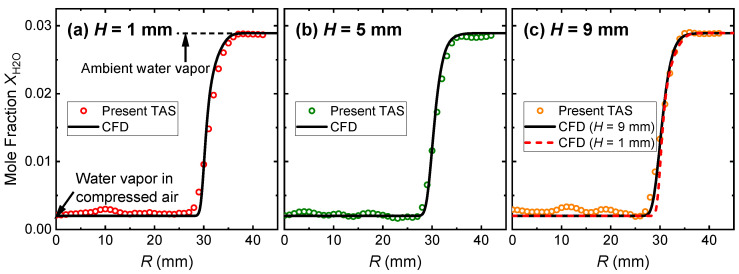
Reconstructed distributions of H_2_O mole fraction X_H2O_ at different heights above the nozzle.

Based on the LOS integrated absorbance *A*_int_ measured equidistantly, the mole fraction of H_2_O *X*_H2O_ was reconstructed radially, as shown in Figure 7. The mole fraction gradients of H_2_O in the shear layer of the laminar jet were well captured. Meanwhile, the concentrations of water vapor in the compressed air and ambient air were also obtained. Particularly, the recovered ambient *X*_H2O_ was very close to the measurement by the electronic thermohydrometer. In the shear layer of the laminar jet at *H* = 1 mm, discrepancies were observed between the present TAS measurement and the CFD simulation, as seen in Figure 7a. However, the variations in the *X*_H2O_ gradients within the shear layer when *H* increased from 1 mm to 9 mm, although subtle, were correctly detected by the present TAS measurements, see Figure 7b,c.

**Figure 8:**
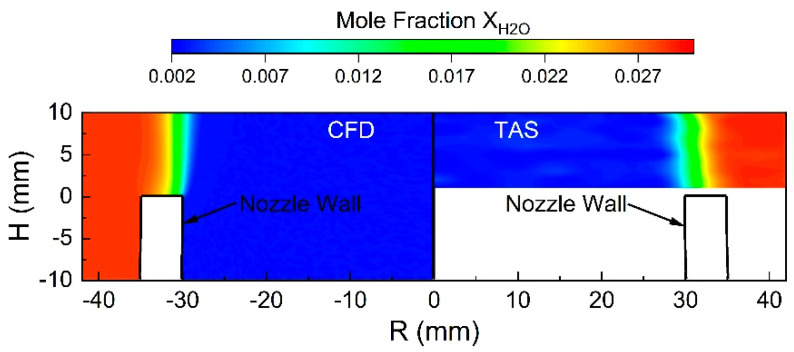
Two-dimensional contour of H_2_O mole fraction distribution. Left, CFD simulation; right, present TAS measurement.

By combining the reconstruction at different heights, a 2D contour of H_2_O mole fraction distribution is displayed in Figure 8, which is in good agreement with the CFD simulation. It can be concluded that TAS has great potential in the research of mass transfer and scalar field of gaseous flows. These results also motivate us to further explore TAS for non-reactive flow measurements in the future, from which we can develop more advanced technologies for complex reactive flows such as combustion. For unsteady flow, a time-resolved TAS measurement system is under construction.

**Figure 9:**
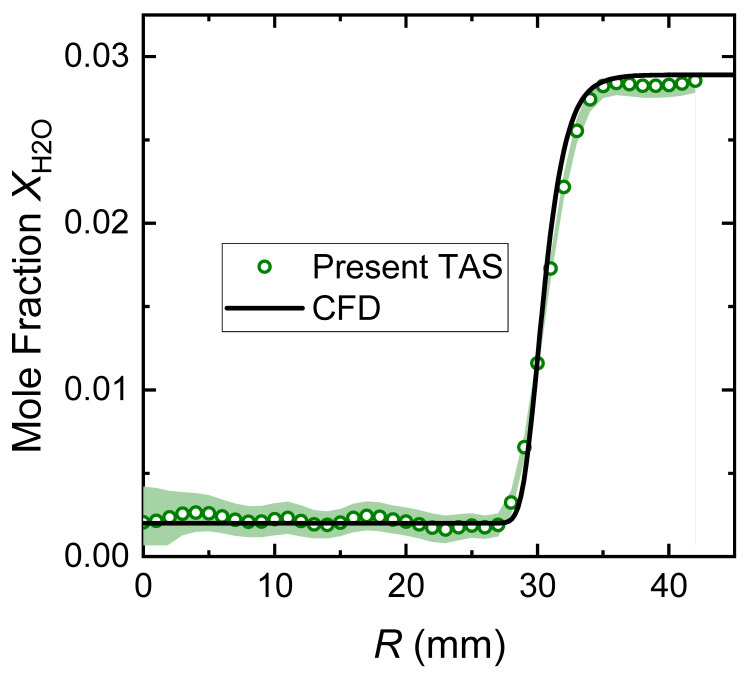
Uncertainty of the present TAS reconstructions of *X*_H2O_ (*H* = 5 mm) in the laminar jet.

Since tomography was applied for the present study, the reconstruction of radial species concentrations is quite sensitive to the error/noise in the integrated absorbance. Although the distribution of the LOS *A*_int_ looks quite symmetric and smooth along the radial direction, the small fluctuation leads to noticeable deviations in the final reconstruction; see Figure 6 and Figure 7. Therefore, the process to obtain *A*_int_, i.e., the Voigt fitting, should account for most of the uncertainty. As we can see from Figure 5b, the uncertainty in *A*_int_ can be estimated as ±2.5%. To evaluate the influence of the uncertainty in *A*_int_ on the final results, we ran 100 random tests with noise-perturbed *A*_int_ data at *H* = 5 mm. On the other hand, the uncertainty in spectral parameters such as linestrength also adds to the final result. As shown in Figure 9, the shaded area represents the 95% confidential interval of the random tests and the ±2.5% uncertainty in linestrength. Overall, the uncertainty of the present TAS measurement is reasonably small (Δ*X*_H2O_ ≈ ±0.0025) considering that we used the ambient H_2_O (*X*_H2O_ = 0.02889) as the absorbing species.

## 4. Conclusions

In the present paper, a detailed absorption spectroscopy measurement for H_2_O transport in a non-reactive laminar jet with an inverse concentration gradient was reported for the first time. The jet was generated using compressed dry air straightened by a honeycomb and a smooth contraction nozzle. To reveal the H_2_O transport in a laminar jet, a tomographic absorption spectroscopy scheme was proposed by using the water vapor in the ambient as the absorbing species, and the absorption line near 1368.598 nm for sensing. One-dimensional tomography was conducted radially at *R* = −42 mm to 42 mm at various heights above the nozzle ranging from *H* = 1 mm to 10 mm. By comparing with the predictions from CFD simulations, it was confirmed that the mole fraction of water vapor *X*_H2O_ can be successfully reconstructed with reasonable uncertainty (±0.0025) by the present optical system. The 2D distribution of *X*_H2O_ for the laminar jet was obtained experimentally using TAS, which is in good agreement with the CFD simulations. Although the signal acquisition and post-processing still need improvements, it was demonstrated that TAS has great potential in the research of mass transfer and the scalar field of gaseous flows. The present method can be further developed to apply to unsteady flows and improved technologies for combustion diagnostics might result from the present serial studies in non-reactive jets. The related work is ongoing and will be reported in the future.

## Figures and Tables

**Table 1:** Main absorption lines selected for Voigt fitting.

Line Number	Frequency (cm^−1^)	Wavelength (nm)	*S* (296 K) (cm^−2^ atm^−1^)	*E*″ (cm^−1^)
1	7306.7396	1368.599	0.027500	325.3479
2	7306.7519	1368.597	0.445359	79.4964
Weighted line ^1^	7306.7470	1368.598	0.472900	93.9700

^1^ Following Wang et al. [32] based on HITRAN2020.

## Data Availability

The data presented in this study are available on request from the corresponding author.
